# Sustained xanthophyll pigments-related photoprotective NPQ is involved in photoinhibition in the haptophyte *Tisochrysis lutea*

**DOI:** 10.1038/s41598-023-40298-z

**Published:** 2023-09-07

**Authors:** T. Lacour, E. Robert, J. Lavaud

**Affiliations:** 1 Ifremer, PHYTOX, PHYSALG, 44000 Nantes, France; 2 Ifremer, PHYTOX, GENALG, 44000 Nantes, France; 3 UMR 6539 LEMAR-Laboratory of Environmental Marine Sciences, CNRS/Univ Brest/Ifremer/IRD, IUEM-Institut Européen de la Mer, Technopôle Brest-Iroise, Rue Dumont d’Urville, 29280 Plouzané, France

**Keywords:** Marine biology, Microbial biooceanography, Ecophysiology

## Abstract

Dynamic xanthophyll cycle (XC) related non-photochemical quenching (NPQd, also called qE) is present in most phototrophs. It allows dissipating excess light energy under adverse growing conditions. Generally, NPQd rapidly reverses for photosynthesis to resume when light intensity decreases back toward optimal intensity. Under certain environmental conditions and/or in some species, NPQ can be strongly sustained (NPQs showing hours-to-days relaxation kinetics). *Tisochrysis lutea* is a South Pacific haptophyte phytoplankton with a strong potential for aquaculture and biotechnology applications. It was previously reported to show a surprisingly low NPQd capacity while synthesizing large amounts of diatoxanthin (Dt), a pigment involved in the XC. In order to better understand this paradox, we investigated the characteristics of NPQ in *T. lutea* under various growth conditions of light and nutrient availability (different photoperiods, low and high light, nutrient starvations). We found a strong NPQs, unmeasurable with usual fluorometry protocols. Along with confirming the involvement of Dt in both NPQd and NPQs (by using the dithiothreitol inhibitor), we highlighted a strong relationship between Dt and the maximum quantum yield of photochemistry (Fv/Fm) across growing conditions and during relaxation experiments in darkness. It suggests that changes in Fv/Fm, usually attributed to the ‘photoinhibitory’ quenching (qI), are simultaneously largely impacted by photoprotective NPQ. The overlap of xanthophyll pigments-related photoprotective NPQ with several other mechanisms involved in the cell response (Photosystem II photoinactivation, changes in pigments composition, and detoxification by antioxidants) to energy unbalance is further discussed. Our findings question both how widespread NPQs is in the global ocean, particularly in nutrient starved environments (oligotrophic waters) and situations (post-bloom), and the use of adapted active fluorescence protocols (i.e. with extended NPQ relaxation period prior to measurement).

## Introduction

The growth of phototrophs is controlled by light energy, temperature and nutrients availability. In both terrestrial and marine environments, growth conditions are highly variable over time and phototrophs must constantly balance energy absorption with its use for growth. Under balanced growth at low irradiance, most of the absorbed energy is generally used for photochemistry. When a stress occurs, such as a light increase, nutrient starvation, temperature drop, cells cannot use all of the absorbed light energy and part of it is dissipated through the so called process of non-photochemical quenching of chlorophyll fluorescence (NPQ). Cells also use multiple antioxidant systems to counterbalance ROS-reactive oxygen species generation and prevent/decrease photoxoidative damages^1,2^. After several hours/days, phototrophs acclimate to the new growth conditions, notably by adjusting light absorption, through the modification of pigment composition and the stoichiometry of photosystems^3^. Some phototrophs, like evergreen plants and polar microalgae, are able to retain almost all their light harvesting capacity, even when growth is completely stopped (e.g. during freezing period) and they dissipate most of the absorbed energy through NPQ^4,5^. On the contrary, other species, like deciduous and annual species, drastically reduce their light harvesting capacity (decrease of pigment content, number of leaves, etc.) to balance the energy budget, and they generally show lower NPQ capacity. However, most of the phototrophs, and especially microalgae, use mixed photoadaptative strategies in accordance to the highly variable aquatic light climate. They are able to concurrently adjust their pigment content and retain their light harvesting capacity during prolonged harsh conditions (high light, low temperature, nutrient starvation), and continuously dissipate the energy in excess through NPQ^5^.

There are three classical, primarily kinetically defined, NPQ components: qE (energy-dependent quenching), qI (photoinhibitory quenching), and qT (state transitions quenching) ^6^. In most eukaryotic phototrophic taxa, the main component of NPQ is qE (see^7–9^ for further details). NPQ-qE is regulated by several partners: the trans-thylakoid ΔpH, the xanthophyll pigments synthesis and cycles (XC), the synthesis of various proteins (e.g. Lhcx), and the spatial reorganization of light-harvesting complexes. Xanthophyll pigments are recognized as key players in NPQ-qE driven thermal dissipation of excess excitation energy in plants and microalgae, but the mechanisms involved are still under debate. A crucial characteristic of NPQ fine tuning versus light energy absorption is its kinetics of induction and relaxation. To fit photochemical efficiency to the environment and optimize growth and productivity, induction and relaxation must be as fast as possible, especially under fluctuating light climates^10^. However, NPQ relaxation kinetic is highly variable across taxa and is dependent on growth conditions. In particular, prolonged low temperatures (overwintering evergreen plants: Verhoeven^11^, Míguez et al.^12^, Demmig-Adams et al.^13^; diatoms: Lacour et al.^14^, Lacour et al.^15^, Wu et al.^16^), high irradiance (diatoms: Lavaud and Lepetit^17^) and general harsh environmental conditions^18,19^ are often associated with sustained NPQ (NPQs, slowly relaxing NPQ, see the review by García-Plazaola et al.^20^). In many studies, NPQs often comprises/overlaps with other types of NPQ named qI, qZ or qH, depending on the authors and the corresponding measurements performed. Indeed, NPQs can be related to several processes, although mainly photosystem II (PSII) photoinactivation and damage (qI^21^) and sustained content in de-epoxidized xanthophylls zeaxanthin (Zx) or diatoxanthin (Dt) (qZ^14,17,20^; qH^22^). Most of the time, the molecular mechanisms underlying NPQs are only partially described and probably multiple (i.e. both qI and qZ involved). NPQs can be considered as photoinhibition because phototrophs exhibiting high NPQs cannot promptly restore maximal photosynthesis rate when more suitable conditions return^13^. It is also described as photoinhibition because it is often measured as so. Indeed, saturating pulse methods conventionally used in (aquatic) photosynthesis are often based on a previous-to-measurement short incubation (5–20 min depending on studies) in darkness or under low light during which NPQ is expected to fully relax, which is, by definition, not achieved when NPQs is present. In this case, the decrease in photochemistry efficiency (i.e. F_v_/F_m_ and ΦPSII) is often attributed to photodamage, and less to sustained Zx or Dt content, when the apparent fluorescence quenching is due to NPQs^14^.

*Tisochrysis lutea* is a motile non-calcified haptophyte. It is widely used to feed mollusk larvae in aquaculture because it is very rich in polyunsaturated fatty acids (particularly DHA). It is very easy to grow in laboratory conditions and it grows fast, in part because it resists to harsh environmental conditions, i.e. *T. lutea* is highly resistant to high irradiance^23^ and it can survive several weeks without nutrients at high irradiance. Previous studies^24,25^ suggested that NPQ is very low in this genus and that other photoprotective mechanisms should explain its strong photoprotective capacity (e.g. alternative and cyclic electron flows). Huang, et al.^24^ reported an increase in slowly relaxing NPQ (hours-time scale, named qNs) in N and P starved cultures of *T. lutea*, and they interpreted it as enhanced PSII photoinhibition. However, *T. lutea* and other Isochrysidales species are highly rich in the xanthophyll pigments diadinoxanthin (Dd) and Dt^26^ particularly under high irradiance^27,28^ and nutrient starvation^29^, suggesting a likely significant involvement of XC-related NPQ-qE in photoprotection. *T. lutea* also shows many LHCx proteins, particularly expressed under high light^30^ that are involved in NPQ-qE in diatoms^31^.

In the present study, we aimed at understanding the physiological ability of *T. lutea* to survive to prolonged harsh conditions. In order to do so, the growth rate, pigment composition, and photochemical parameters were measured in cultures grown at three light intensities (40, 280 and 550 µmol photons m^−2^ s^−1^), under several kind of nutrient stress (N-Nitrogen, P-Phosphorus and vitamin B_12_ starvations in batch cultures) under continuous illumination, and under N sufficient versus starved cultures under light:dark exposure. We additionally undertook several targeted experiments to study some specific processes (xanthophyll synthesis, NPQ relaxation) likely crucial to *T. lutea* survival.

## Materials and methods

### Growing and experimental conditions

Unialgal cultures of *Tisochrysis lutea* (CCAP 927/14) were grown in pre-filtered Walne’s medium at 23 °C, illuminated by white fluorescent tubes (OSRAM FQ 54W/965 HO LUMILUX Cool Daylight) and gently aerated through 0.3 µm-pore-filters. Irradiance was measured with a spherical QSL-100 quantum sensor (Biospherical Instruments, San Diego, CA, USA) in front of the culture vessel. *T. lutea* were grown under two lighting modes (continuous versus Light: Dark illumination), under a range of growth light intensities and under several nutrient regimes (N, P and vitamin B_12_ starvations) (Table 1). Nutrient replete cultures were acclimated to continuous (photoperiod 24L: 0D) high (HL, 530 µmol photons m^−2^ s^−1^), medium (ML, 300 µmol photons m^−2^ s^−1^), and low (LL, 40 µmol photons m^−2^ s^−1^) irradiances, and to L:D illumination (photoperiod 12L:12D, daily mean and maximal growth irradiance:125 and 450 µmol photons m^−2^ s^−1^, respectively). The irradiances at which cultures were acclimated is from hereafter called “growth irradiance”. Growth conditions were maintained semi-constant by diluting cultures with fresh medium once a day or every 2 days^32^. N, B_12_ and P starvations were achieved after 15 days in media lacking N, B_12_ and P, respectively, when growth had decreased to 0 d^−1^. Semi-continuous cultures, were sampled when cultures reached steady state (sensu MacIntyre and Cullen^32^, i.e. after cells were completely acclimated to the growth conditions after a minimum of 10 cell generations). We performed daily measurements of the growth rate, cell diameter and active chlorophyll *a* (Chl *a*) fluorescence to monitor the acclimation of the cultures to the growth conditions^33^. Cultures were also sampled for measuring cell number, pigment composition, and active Chl *a* fluorescence. The L:D illumination condition was sampled at high frequency during one light cycle (32 h) to understand the diel variations of the photophysiological properties in *T. lutea*.

**Table 1:** Conditions of the different *Tisochrysis lutea* cultures performed in this study.

Growth conditions	Culture mode	Irradiance (µmol photons m^−2^ s^−1^)	Growth rate (d^−1^)	Fv/Fm^1 min^	Fv/Fm^15 min^	rETRm	NPQ_d_^m d^
LL	Semi-continuous	40	0.92 ± 0.02	0.64 ± 0.00	0.66 ± 0.00	242.18 ± 3.67	0.21 ± 0.02
ML	Semi-continuous	300	1.16 ± 0.06	0.55 ± 0.00	0.60 ± 0.01	324.98 ± 19.35	0.30 ± 0.01
HL	Semi-continuous	530	1.39 ± 0.05	0.33 ± 0.01	0.42 ± 0.00	288.82 ± 8.05	0.17 ± 0.06
N starved	Batch	530	0	0.23 ± 0.01	0.35 ± 0.02	NA	NA
B_12_ starved	Batch	530	0	0.23 ± 0.01	0.42 ± 0.01	214.33 ± 15.36	0.72 ± 0.18
P starved	Batch	530	0	0.24 ± 0.04	0.37 ± 0.02	165.24 ± 8.17	0.50 ± 0.23
N replete, L:D illumination	Semi-continuous	Daily mean : 125 Max : 450	0.77 ± 0.04	*	*	*	*
N starved, L:D illumination	Batch	Daily mean : 125 Max : 450	0	*	*	*	*

*Fv/Fm (15 and 1 min), rETRm and NPQd show diel variations (see text and Fig. 3).

LL: Low Light; ML: Medium Light; HL: High Light, Fv/Fm^1min^ and Fv/Fm^15min^: apparent maximum quantum yield of PSII after 1 min and after 15 min respectively; rETRm: relative electron transport rates; NPQ_d_^m^: maximum dynamic Non Photochemical Quenching (see “Methods” section).

### Targeted experiments

Culture samples were incubated under two light conditions to perform a dark relaxation experiment and a high light shift experiment.

#### Dark relaxation experiment

To document the relaxation kinetics of NPQs, we incubated in complete darkness cultures previously acclimated to 1-continuous light and N, P, B_12_ starvations, and 2-L:D illumination and N replete or starved conditions. We monitored pigment composition and active Chl *a* fluorescence.

#### High light shift experiment

Triplicates of culture samples previously under P starvation were treated with and without dithiothreitol (DTT) and incubated 20 min in the dark. DTT dissolved in milliQ water was added from a freshly prepared 50 mM stock solution to a final concentration of 500 µM, a concentration high enough to inhibit all Dd de-epoxidation in Dt^34^. Cultures samples were incubated at high irradiance during 60 min and then in darkness during 60 min. We monitored pigment composition and active Chl *a* fluorescence.

### Cell number and pigment analysis

*T. lutea* cells were counted and sized (cell volume) using a Beckman Multisizer 4 Coulter Counter (Miami, US). For pigment analysis, an aliquot of algal culture (5 mL) was filtered onto GF/F glass-fiber filters (Whatman R©), immediately flash-frozen in liquid nitrogen and stored at − 80 °C until analysis by High Performance Liquid Chromatography (HPLC). Pigments were extracted from the frozen filters by sonication in 2 mL of 95% acetone and filtered with PTFE syringe filters (pore size 0.2 µm) into HPLC vials. The filtered acetone extracts were analyzed by HPLC-UV-DAD (series 1200 HPLC–UV-DAD; Agilent Technologies) using a guard column and an Eclipse XDB-C8 reverse phase column (150 mm × 4.6 mm, 3.5 μm particle size; Agilent Technologies) following the method described by Van Heukelem and Thomas^35^. Briefly, solvent A was 70:30 MeOH: H_2_O 28 mM ammonium acetate, and solvent B was pure MeOH. Gradient elution was the same as described in Van Heukelem and Thomas^35^. Concentrations of Chl *a* and *c2*, and of carotenoids fucoxanthin (Fuco), diatoxanthin (Dt), diadinoxanthin (Dd), echinenone (Echin) and beta-carotene (β-Car) were determined from DHI (Denmark) standards. The xanthophyll de-epoxidation state (DES in %) was calculated as Dt/(Dd + Dt) × 100^10^.

### Growth rates

Biomass growth can be estimated from any index of biomass, such as cell number, Chl *a*, carbon (C), or N variation rates^32^. The specific variation rate of a compound *i* having a bulk concentration C_i_ can be estimated (at time t_2_) from two discrete data measurements performed at times *t*_*1*_ and *t*_*2*_ following:

1 $$\mu_{i} \left( {t_{2} } \right) = \frac{{\ln \left( {\frac{{C_{i} \left( {t_{2} } \right)}}{{C_{i} \left( {t_{1} } \right)}}} \right)}}{{t_{2} - t_{1} }}\quad \left( {{\text{d}}^{ - 1} } \right)$$

In the following text, µ_C_, µ_Cell_, µ_N_ and µ_Chl*a*_ are the specific rates of variation of carbon, cell, nitrogen and Chl *a* respectively. µ_Cell_ is the cell division rate.

### Active Chl a fluorescence measurements

Variable Chl *a* fluorescence measurements were performed using a Phyto-PAM (Pulse Amplitude Modulated) fluorometer (Walz GmbH, Effeltrich, Germany). The fluorometer applies a saturating pulse (800 ms pulse of 4000 µmol photons m^−2^ s^−1^) to the incubated sample and generates a fluorescence (detected at 680 nm) induction curve that can be used to estimate the minimum fluorescence (F0 when dark-acclimated), the steady-state fluorescence at light (Fs) and the maximum fluorescence (Fm when cells are dark-acclimated and Fm’ when light-acclimated). Rapid light curves (RLC) were recorded by applying 21 steps of increasing light intensity up to 2064 µmol m^−2^ s^−1^ with a respective duration of 10 s.

We estimated the apparent maximum quantum yield of PSII after 1 min (Fv/Fm^1min^) and after 15 min (Fv/Fm^15min^) of dark acclimation as follows (see Van Kooten and Snel^36^):

2 $${\text{Fv}}/{\text{Fm}} = { }\frac{{{\text{Fm}} - {\text{F}}0}}{{{\text{Fm}}}}$$

Fv/Fm^1min^ was measured in order to compare the pigment composition (also determined after 1 min in darkness) and the photochemical properties in the same conditions.

Under stressfull conditions, *T. lutea* exhibits a very strong sustained NPQ (NPQs, i.e. NPQ is not fully relaxed after 15 min in darkness), which prevents reliable NPQ measurements. We thus separated NPQ into two components, sustained and dynamic NPQ, respectively named NPQs and NPQd^16^. NPQd and NPQs were calculated as follows:

3 $${\text{NPQd }} = { }\frac{{{\text{Fm}} - {\text{Fm}}^{\prime } }}{{{\text{Fm}}^{\prime } }}$$

4 $${\text{NPQs }} = { }\frac{{{\text{Fm}}^{{\text{d}}} - {\text{Fm}}}}{{{\text{F}}_{{\text{m}}} }}$$

where Fm^d^ is Fm from cells incubated in the dark for several hours (see ‘Dark relaxation experiment’ above). In most of the growth conditions examined here, NPQs is certainly underestimated because NPQ was not fully relaxed even after several hours in the dark (see the “Results” section).

RLCs also allowed computing relative photosynthetic electron transport rates (rETR) as follow:

5 $${\text{rETR}} = { }\frac{{F_{m}{\prime} - {\text{Fs}}}}{{F_{m}{\prime} }}{ } \times {\text{E}}$$

where E is the incubation irradiance.

rETRm and NPQd^m^ were estimated by fitting the equation of Platt et al.^37^ (without the photoinhibition parameter β) to the experimental values.

## Results

### Growth rate, photochemical properties and pigments versus growth conditions under continuous 24 h Light:0 h Dark illumination

The growth rate of *T. lutea* increased with growth irradiance from 40 to 530 µmol photons m^−2^ s^−1^ showing that at least at 300 µmol photons m^−2^ s^−1^ growth was not completely light-saturated (Table 1). Fv/Fm^1min^ and Fv/Fm^15min^ decreased with increasing growth irradiance. The apparent maximum dynamic NPQ (NPQd^m^) was very low (mean value: 0.23 ± 0.07, dimensionless). Nutrient starved cells showed low Fv/Fm^15min^ (0.35 to 0.42) and higher NPQd^m^ (mean value: 0.64 ± 0.12) than nutrient replete cells (Mann–Whitney Rank Sum Test, *P* = 0.002). Starved cells showed relatively high rETRm (mean value = 158 ± 59 versus 285 ± 41 under replete conditions) although these cells were not able to grow (µ = 0 d^−1^).

**Figure 1:**
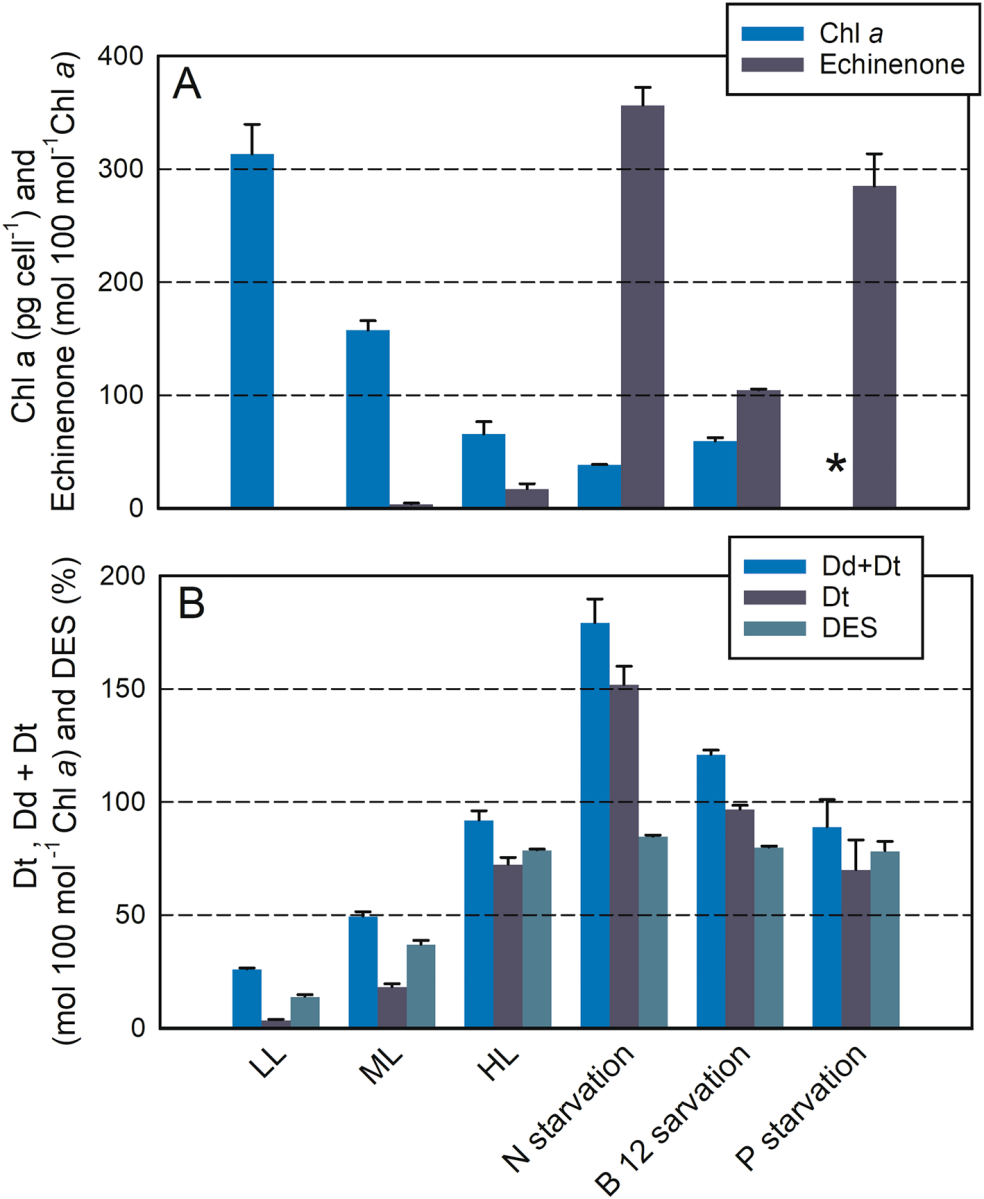
(**A**) Chl *a* cell^−1^ and echinenone/Chl *a*, (**B**) (Dd + Dt)/Chl *a*, Dt/Chl *a*, de-expoxidation ratio (DES) in *Tisochrysis lutea* grown under various light and nutrient conditions (see Table 1). Each data point is the mean of 2 to 3 independent cultures, error bars represent standard deviations. *Note that in A, Chl *a* cell^−1^ is not available under P starvation.

**Figure 2:**
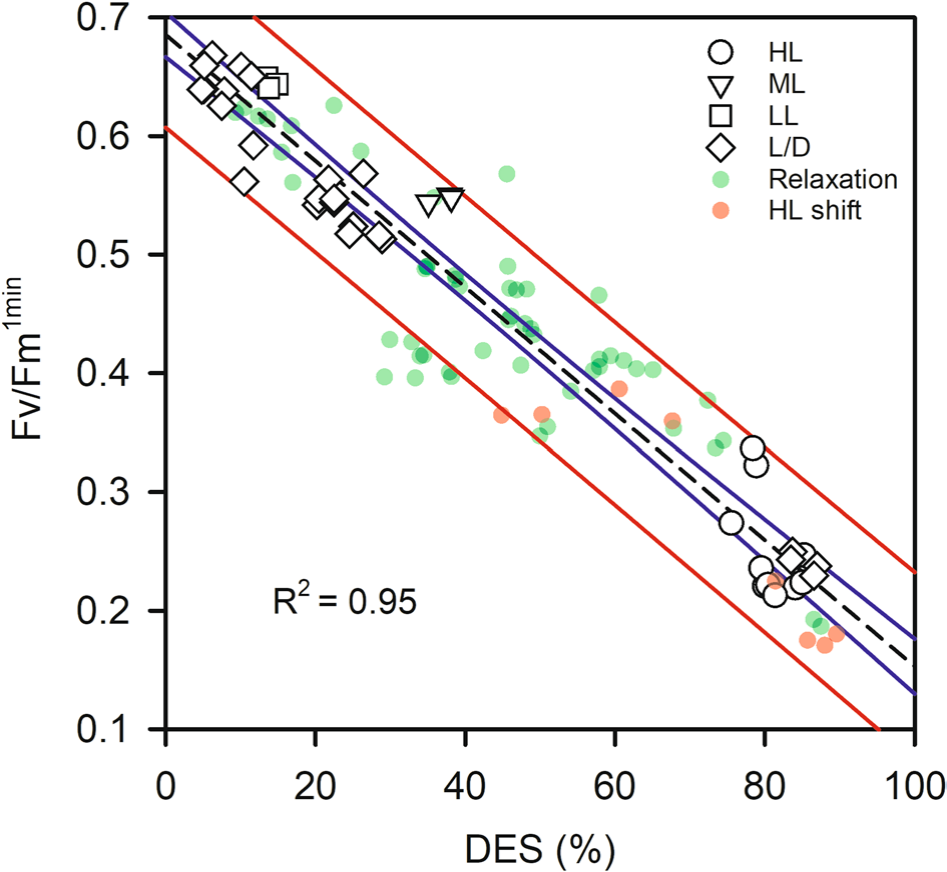
Relationship between Fv/Fm^1min^ and the de-expoxidation ratio (DES) under the diverse growth conditions studied here. The dataset has been separated according to the growth irradiance (HL: nutrient replete, N star, P star and B_12_ star at 530 µmol photons m^−2^ s^−1^; ML: nutrient replete at 300 µmol photons m^−2^ s^−1^; LL: nutrient replete at 40 µmol photons m^−2^ s^−1^). We also separated the L:D, the relaxation and the HL shift experiments. Linear regression (fitted on the full dataset) is shown with 95% confidence and prediction intervals, and corresponding R^2^ are provided.

As expected, Chl *a* cell^−1^ decreased with growth irradiance (Fig. 1A). Chl *a* cell^−1^ under N starvation and B_12_ starvation were lower than under HL. Beyond the Chl *a* content per cell, *T. lutea* also strongly modulated several pigments versus Chl *a*: the xanthophyll cycle pigments (diadinoxanthin (Dd) and diatoxanthin (Dt)) and the echinenone. Dd, Dt and the de-epoxidation ratio (DES) increased with growth irradiance (Fig. 1B). Dd + Dt reached up to ≈ 1.5 times Chl *a* under N starvation. DES reached a maximum value of ca. 80% under several conditions (HL, N star, B_12_ star, P star), i.e. in cells cultured at 530 µmol photons m^−2^ s^−1^. In order to understand the possible involvement of Dt in the decrease in the quantum yield of photochemistry (Fv/Fm), we computed the relationship between Fv/Fm^1min^ and DES across conditions and highlighted a linear relationship of R^2^ = 0.95 (Fig. 2). The echinenone was undetectable or low under replete conditions, even under HL (Fig. 1A). On the contrary, cells under prolonged N, B_12_ and P starvation showed around 1.5, 1 and 3 times more echinenone than Chl *a*, respectively.

### Growth rate, photochemical properties and pigments versus growth conditions under 12 h Light:12 h Dark illumination

Under L:D illumination and N replete conditions, the two duplicate cultures showed very similar daily-averaged cell division rates of 0.77 ± 0.04 d^−1^, corresponding to 1.11 divisions per day (Table 1). The light signal synchronized the cell population: most of the cells divided during the night (cell density increase during the night, Figure S1A), mean cell volume increased during the day (Figure S1B) and decreased during the night due to cellular somatic growth (C fixation) during the day (Figure S1C), and the cell division and C consumption (respiration) at night. Pigment synthesis only occurred during the light period with a maximum at midday (see Chl *a* and xanthophylls contents in Figure S1C, D). Under N starvation, cell division (Figure S1A) and N assimilation stopped (Figure S1D) and C fixation during the day decreased drastically (Figure S1C). Pigment synthesis also decreased considerably but continued to oscillate with a lower amplitude (Figure S1E, F).

**Figure 3:**
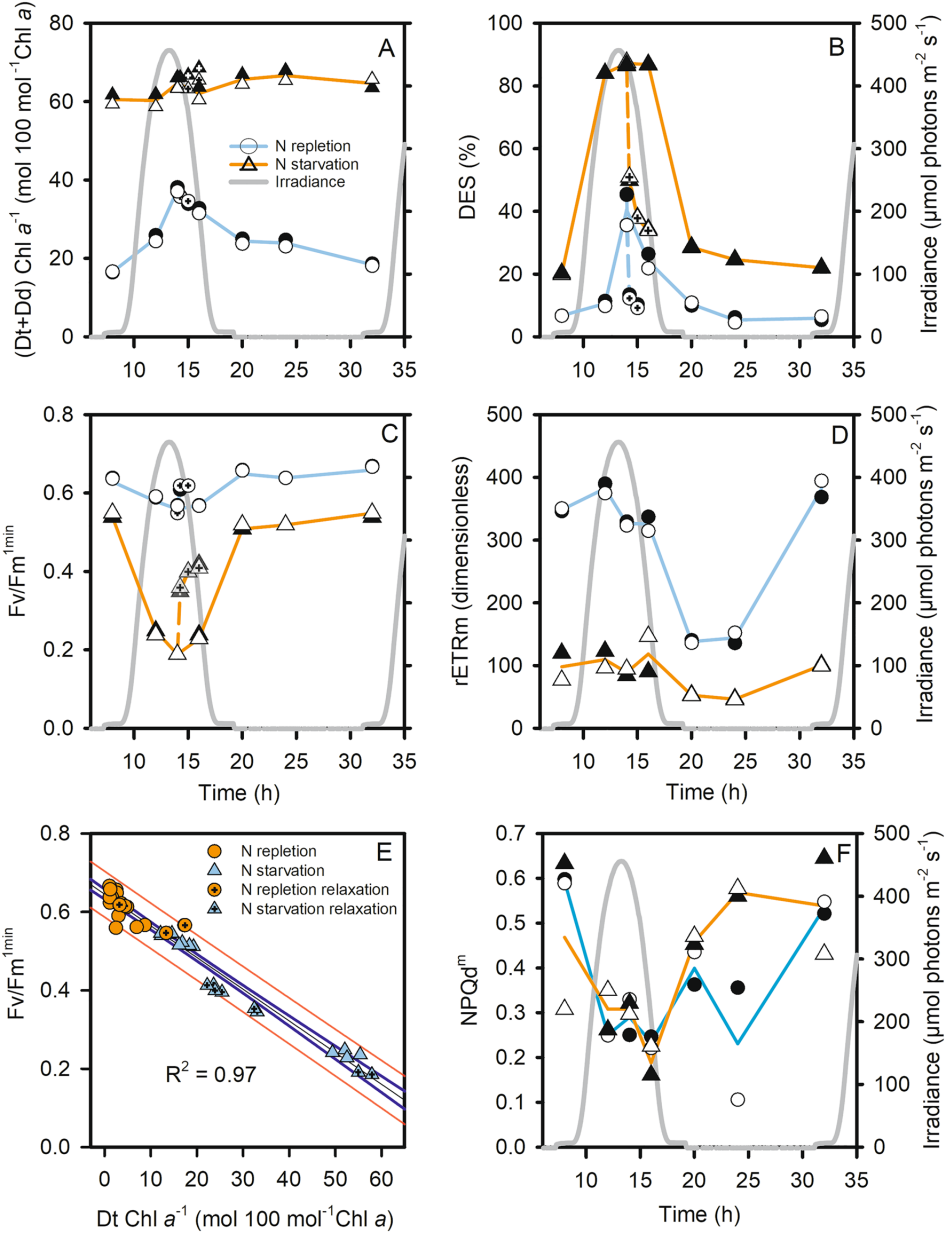
Time dependent change in (Dd + Dt)/Chl *a* (**A**), de-expoxidation ratio (DES = Dt/(Dd + Dt) × 100) (**B**), Fv/Fm^1min^ (**C**), rETRm (**D**) and NPQ_d_^m^ (**F**) during a diel cycle and relationship between Fv/Fm^1min^ and Dt/Chl *a* (**E**) in N-sufficient (circles) and N-starved growth (triangles). In A, B, C, and E, crossed symbols represent the two replicates of the relaxation experiments (incubation in darkness, see the “Methods” and “Results” section). In A, B, C, D and F, a line passes through the mean of the two replicates. The grey line represents growth irradiance.

Under N replete conditions, the xanthophyll pigment pool (Dd + Dt) also showed a diel variation with a maximum value at 14:00 (Fig. 3A) and the DES showed a strong diel cycle with a maximum value at 14:00 (≈ 40%) (Fig. 3B). Under N starvation (Dd + Dt)/Chl *a* was much higher (≈ 60 mol 100 mol Chl *a*^−1^) and without diel oscillations. On the contrary, the diurnal accumulation of Dt was maintained and with much higher amplitude, reaching more than 80% of de-epoxidation at 14:00 and a minimum DES > 10% during the night (Fig. 3B). Fv/Fm^1min^ showed an opposite pattern to the DES, with a slight decrease during the day under N replete conditions, and a sharp decrease under N starvation (Fig. 3C). As a consequence, there was a strong relationship between Fv/Fm^1min^ and both Dt (Fig. 3E) and DES (diamonds in Fig. 2). Interestingly, rETRm was high during the light period, i.e. when Fv/Fm^1min^ was low (see also Fv/Fm^15min^ in Figure S2A) and it decreased overnight (Fig. 3D). Under N starvation, rETRm was 3–4 times lower and showed the same diel pattern but with lower amplitude. NPQd^m^ remained relatively low during the L:D cycle and did not change with the N status of the cells (Fig. 3F). We did not find any significant relationship between the Dt content and NPQd^m^ (Figure S2B).

### Relaxation kinetics of the sustained NPQ (NPQs): the role of Dt

**Figure 4:**
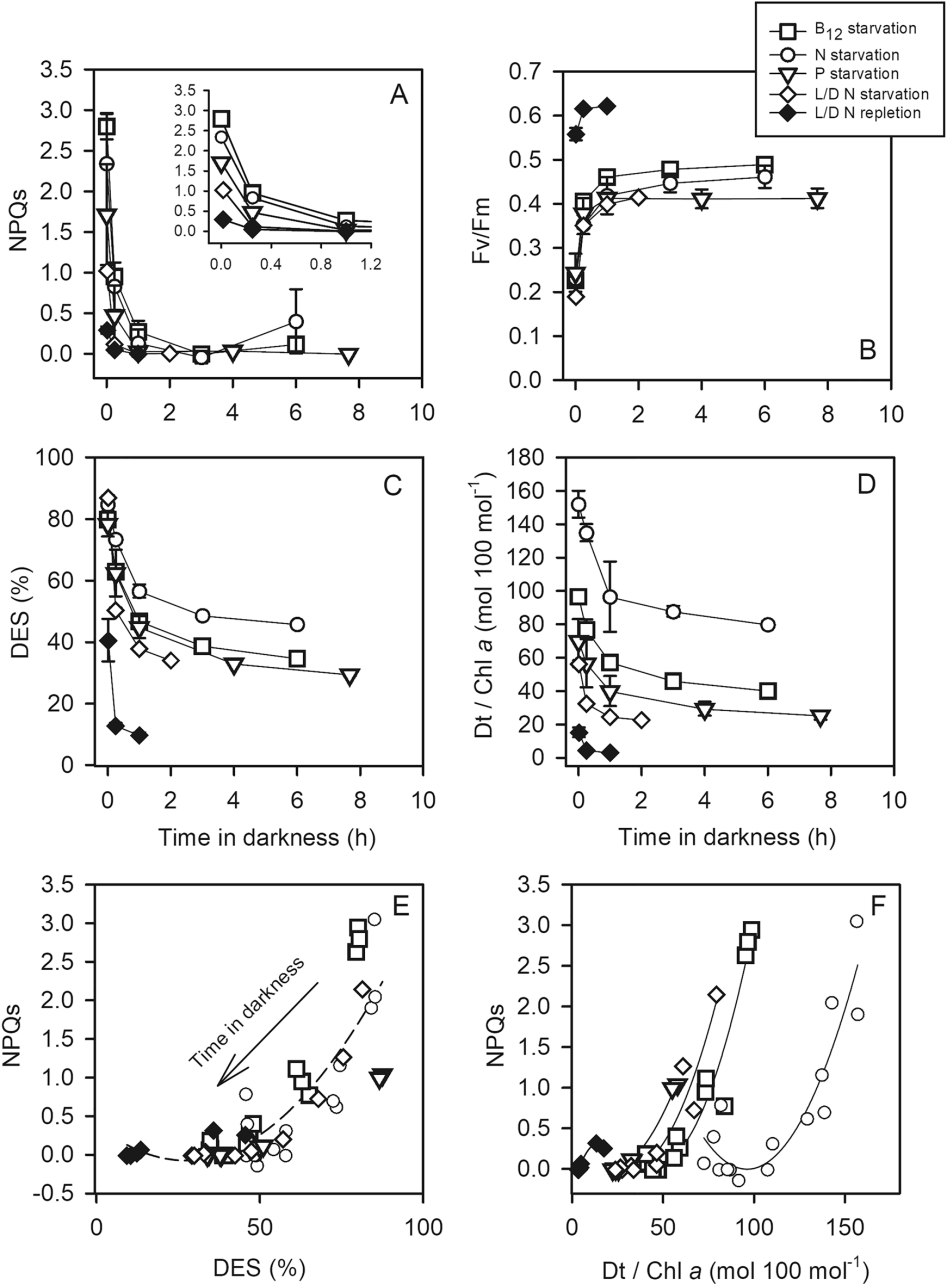
Darkness time dependent change in (**A**) Fv/Fm, in (**B**) the sustained NPQ (NPQs), in (**C**) de-expoxidation ratio (DES = Dt/(Dd + Dt) × 100) and in (**D**) Dt/Chl *a* in *T. lutea* cells under B_12_, N, P starvation, and under L/D illumination (t_0_ at noon). (**E**) and (**F**): relationship between DES, Dt/Chl *a* and NPQs, respectively; data are from panels (**A**–**D**). Each data point is the mean of 2 (L/D cycles) or 3 independent cultures, error bars represent standard deviations.

The virtual absence of apparent NPQd in cells containing very high amount of Dt and showing low Fv/Fm suggests that the protocol for NPQ measurements was inappropriate. Indeed, in the presence of NPQs, even after 15 min of darkness acclimation, Chl *a* fluorescence can still be strongly quenched^38^. We thus proceeded with longer (several hours) dark incubations to look at the epoxidation kinetics of Dt and the relaxation kinetics of Chl *a* fluorescence quenching under growth conditions for which the DES was high (around 80%): continuous HL + B_12_, N and P starvations, L:D cycle + N starvation, and we computed NPQs as described in the “Methods” section. NPQs was higher in the nutrient starved cultures (Fig. 4A) and it decreased quickly during the first hours in darkness and then remained relatively constant. Fv/Fm showed an opposite pattern with a strong increase during the first hours followed by a more moderate increase or stagnation thereafter (Fig. 4B). In parallel, the DES and Dt content of nutrient starved cells slowly decreased, taking about 2 h to decrease by 50% (Fig. 4C,D). After several hours in darkness (6–8 h), the amount of Dt was still very high. It suggests that NPQs was possibly not fully relaxed, and that values in Fig. 4A are possibly underestimated. When plotted against DES, NPQs showed a unique curvilinear relationship highlighting the strong link between NPQs and sustained Dt (Fig. 4E). A strong inverse linear relationship was also observed between DES and Fv/Fm (green circles in Fig. 2). When plotted against Dt, NPQs showed curvilinear relationships too (Fig. 4F).

**Figure 5:**
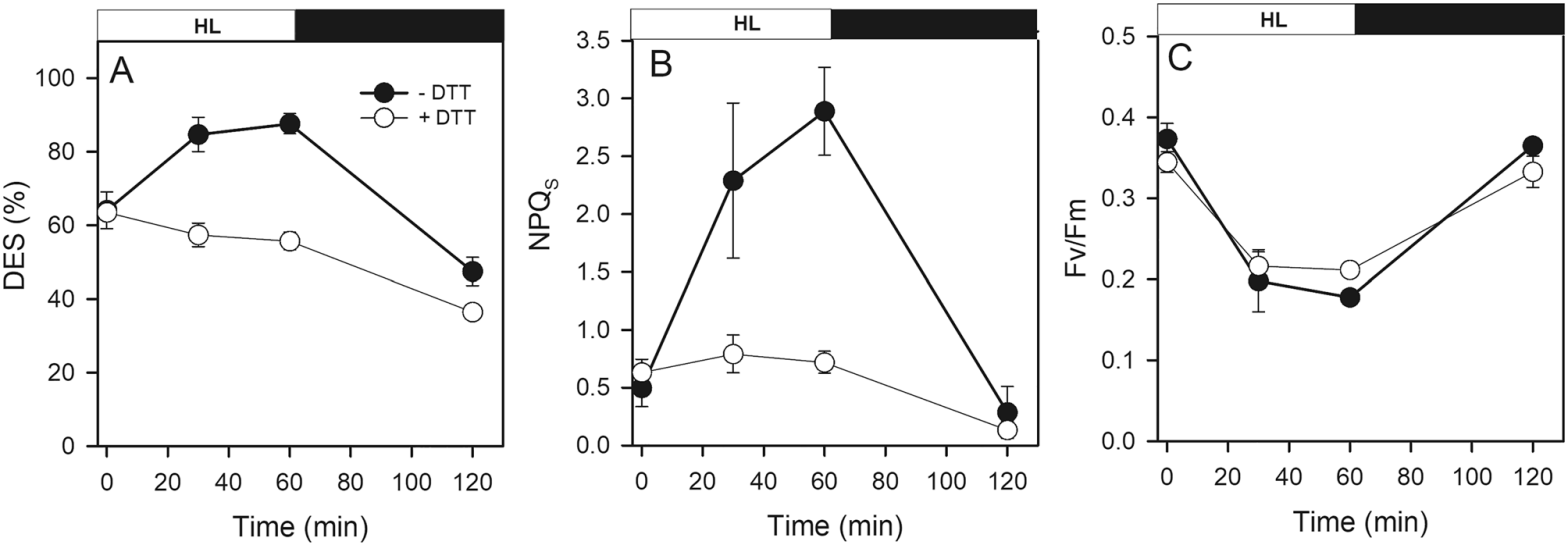
Time dependent change in (**A**) DES, (**B**) NPQs, and (**C**) Fv/Fm in *T. lutea* cells under P starvation during a 60 min light (700 µmol photons m^−2^ s^−1^)/60 min dark experiment in the presence (open circles) and absence (closed circles) of DTT. Each data point is the mean of 3 independent cultures, error bars represent standard deviations.

To confirm the contribution of Dt molecules in NPQs in cells with a high Dt content (i.e. starved cultures), we incubated P starved cultures during 1 h at higher irradiance (700 µmol photons m^−2^ s^−1^) followed by 1 h recovery in darkness with and without dithiothreitol (DTT), a well-known inhibitor of the Dd de-epoxidase enzyme (see the “Methods” section). DTT successfully prevented the synthesis of new Dt molecules from Dd de-epoxidation due to high light exposure and totally abolished the increase in DES and NPQs observed in cells without DTT (Fig. 5A,B). Interestingly, we did not observe any differences in Fv/Fm between DTT treated and untreated cells (Fig. 5C).

## Discussion

Persistent nutrient stress coupled to high irradiances are widespread conditions in the oceans, particularly in post-bloom situations and in oligotrophic gyres all year round, when mixing depth and nutrient concentrations are low^39^. In order to better understand the phytoplankton capacity to survive prolonged periods under harsh conditions, we grew the haptophyte *Tisochrysis lutea* under several light levels and nutrient stresses, typically found in its natural habitat. *T. lutea* is encountered at low density in all seas^40^ and the strain we used in this study (CCAP 927/14) has been isolated in Tahitian oligotrophic waters (French Polynesia) where nutrients are often scarce and irradiance is high.

### Light energy capture

Phytoplankton photosynthesis is regulated in response to the light availability and the demand for photosynthates (sink activity) to sustain cell growth and division. Under a strong nutrient stress, cell division rate decreases, leading to a decrease in the demand for photosynthates. To prevent oxidative damage under such conditions, phytoplankton generally decrease their light absorption capacity^33,41^. This behavior is particularly crucial when the light availability is substantial and in excess of photochemical capacity even for low irradiances. As expected, we measured much lower pigment content at high irradiance and in nutrient starved cells in comparison to nutrient replete cells (see Chl *a* content in Fig. 1), suggesting a strong decrease in absorption capacity. This major photoacclimative process has been widely described in both monocultures^3,42^ and natural phytoplankton communities^43–45^. Halsey et al.^46^ showed that, under balanced growth in chemostat, i.e. after complete acclimation to the nutrient limitation, most of phytoplankton species adjust their absorption capacity in proportion to their ability to grow. Under starvation (µ = 0 d^−1^, very low sink activity), cells reduce drastically their absorption capacity but a significant proportion of absorbing pigments remain, leading to an energy input that has to be channeled toward bioenergetic routes alternative to photochemistry.

### Dynamic NPQ

Microalgae use a cascade of photoprotective mechanisms to safely channel the energy absorbed in excess^6^. One of the first steps is the photoprotective dissipation of energy as heat within the light harvesting complex (LHC), a process which is generally assessed using saturating pulse methods and called Non-Photochemical Quenching of Chl *a* fluorescence (NPQ)^6^. Using classic protocols (such as Rapid Light Curve, RLCs), we measured low dynamic NPQ in nutrient replete cultures even under strong, continuous and saturating irradiance (0.17 at 530 µmol photons m^−2^ s^−1^ under a 24 h L: 0 h D photoperiod) suggesting that NPQ is apparently poorly involved in *T. lutea* photoprotection at high irradiance. The same conclusion has been previously reported by several authors^24,25^. Under nutrient starvation, NPQd^m^ was nevertheless higher but remained low (< 0.75) in comparison to other microalgae^5^.

### Xanthophyll cycle pigments and their involvement in NPQ

In parallel, we measured very high content of the xanthophyll pigments diatoxanthin (Dt) and diadinoxanthin (Dd) (Fig. 1B) particularly under high light and nutrient stress. Dt and Dd, which has been reported in *T. lutea* before^27–29,47^ are generally involved in the so-called xanthophyll cycle (XC). This cycle, found in the main algal classes Bacillariophyceae (diatoms), Xanthophyceae, Haptophyceae, and Dinophyceae^9,48^ consists of one de-epoxidation step that converts Dd into Dt within the LHC antennas to convert them in a heat-dissipating state. The very high level of de-epoxidation (DES, Fig. 1B) contrasted with the very low NPQd^m^ measured using PAM fluorimetry, suggesting that either Dt was not involved in NPQ in *T. lutea*^5,49^ or the protocol used here did not allow to reliably estimate the full amplitude of NPQ under our growth conditions.

Adams and Demmig-Adams have repeatedly demonstrated^50^ in land plants that the assessment of NPQ by active fluorometry is quick, easy and non-destructive, but has several pitfalls to be considered, especially for ecophysiological purposes. One of the main problems resides in the assumption that the dark (or low light) incubation (generally between 5 and 20 min) before measurement allows to completely dissipate NPQ, and therefore to accurately estimate Fm (the maximal level of unquenched Chl *a* fluorescence). For example, sun- and cold-adapted plants and trees often show sustained XC pigments related-NPQs, i.e., that remain locked-in even after a long recovery period in darkness or low light^11–13^. In several microalgae species, similar retention of de-epoxidized zeaxanthin or Dt, and related NPQs, under different conditions of light and temperature, has been reported^14,16^, making NPQ measurement with classic fluorimetry protocols problematic.

### Xanthophyll cycle-related ‘photoinhibition’

*T. lutea* exposed to prolonged high light and nutrient stress shows low apparent maximum efficiency of photochemical energy conversion (Fv/Fm^15min^, Table 1). Low Fv/Fm has already been observed in monocultures under similar conditions^51,52^ and in the field^53^. At global scale, Falkowski and collaborators revealed that the average quantum yield of photochemistry in the world ocean surface is approximately 0.35 (i.e. half of the microalgae maximum), and suggested that it was caused by the scarcity of inorganic nutrient in surface waters where irradiance is high^54^. Sustained depression in PS II efficiency (or O_2_ production quantum yield) is often interpreted as impaired or non-functional PSII reaction centers, and/or uncoupled photosynthetic LHC antenna, and generally called “photoinhibition”. However, only few studies have been performed^21^ to describe the molecular mechanisms involved in this widespread "photoinhibition" phenomenon (~ 60% of the photosynthetically active quanta absorbed by phytoplankton globally, according to^54^). In *T. lutea*, Fv/Fm strongly correlated with DES for all the growth conditions tested (Fig. 2) which is consistent with Dt playing a role in the decrease in photochemical efficiency.

### XC-related photoinhibition under light:dark photoperiodic alternance

Most studies on the effect of growth conditions on photoprotection were performed under a stable intensity during the light period or even under continuous light (i.e. 24 h Light:0 h Dark with constant light)^15,28^. However, natural light cycle regulates most of the cellular processes of all photosynthetic organisms in the ocean, including cell division, pigment synthesis, nutrient capture and carbon fixation^55,56^. Phototrophs need to find a compromise between absorption capacity to maximize light energy capture when irradiance is low or moderate (morning, evening) and photoprotection capacity to survive through the midday light maximum. We observed diel oscillation of Dt, DES and Fv/Fm and correlation between these parameters in accordance to what is often observed at sea surface^57,58^ and also in land plants^59^.

Under nutrient replete conditions, the amount of Dt + Dd, which represents the potential for XC-related NPQ^5^, was synchronized by the light:dark cycle. Interestingly, we observed a negative xanthophyll-specific growth rate (µ^Dt+Dd^, Figure S1F) during the afternoon, when light pressure decreased, showing that a part of the XC pigments were converted into other compounds, possibly the light harvesting carotenoid fucoxanthin^60^. It would be an efficient way to convert photoprotection capacity into light absorption capacity when the irradiance decreases over hours. Under N starvation (cell division stopped), the amount of Dt + Dd was much higher, as observed under continuous illumination, and remained high throughout the light:dark cycle. In both replete and starved conditions, DES oscillated with light, with a much higher amplitude under N starvation when the need for photosynthates is much lower than under N repletion. Consequently, for a same light intensity, N starved cell have to dissipate a greater proportion of the absorbed light energy. Taking both conditions together, we found a very strong relationship between Fv/Fm, Dt (Fig. 3E) and DES (Diamonds in Fig. 2), which, in addition to clues presented before, strongly suggests an involvement of Dt in the decrease in PSII efficiency.

Under starvation, XC pigments are the only pigments which concentration continues to change with light intensity, showing that the activity of the de-epoxidase/epoxidase enzymes were not restricted. However, it is important to point out that the XC-related NPQ was not completely relaxed overnight, especially under N starvation, when a quarter of the Dt was still retained by the end of night. The NPQ potential measured using RLCs after 15 min recovery in darkness (NPQ_d_^m^, Figure S2) does not correlate with the Dt content (see Fig. 3F) nor DES. This absence of correlation shows that this method is not appropriate to estimate the dynamic XC-related NPQ under light:dark illumination in this species, even at moderate irradiance (mean growth irradiance:125 µmol photons m^−2^ s^−1^) and under replete conditions.

### Long term sustained NPQ

In plants, two different types of sustained NPQs were described: qZ, which is PsbS- and ΔpH-independent and requires zeaxanthin, and the newly-termed qH which is independent of the zeaxanthin synthesized from violaxanthin^22^. qZ is particularly prevalent in evergreens^61^ which maintain a strong light absorption capacity even when growth conditions are unfavorable. Phytoplankton also maintain absorption capacity in excess of photochemical needs under long term unfavorable conditions and use NPQs to dissipate excess energy. NPQs is generally related to Dt (or zeaxanthin) molecules and it shows very low quenching efficiency in comparison to NPQd that is observed during short term increase in light intensity^5^. In the same way, in *T. lutea*, long term NPQs relaxes very slowly (Fig. 4, hours time scale) and is clearly related to Dt. Kinetic overlap of photoinactivation and downregulation of PSII has been described before in microalgae, particularly in diatoms^6,31^. We showed here that, in the haptophyte *T. lutea,* particularly under nutrient deficiency, qZ is an important contributor to NPQ and should not be confused with qI.

Quenching efficiency is difficult to estimate because NPQ is largely underestimated as we were not able to estimate the true Fm under our experimental conditions. Also, NPQd induced using RLCs is always relatively low and only poorly related to Dt. It is contrary to what is observed in diatoms grown at low irradiance, which possess a strong XC-related NPQd, particularly when NPQs is low^14^. Overall, we do not know much about the molecular players of this slowly relaxing ‘photoinhibitory-like’ NPQs process. Thylakoid membrane proteins called Lhcx have a key role for NPQ induction in diatoms^31^. *T. lutea* possesses 12 *lhcx* genes which specific role in the different components of NPQs is not known^30^. Furthermore, the regulation of the xanthophyll cycle enzymes^62^ allowing such locked-in NPQ remain to be investigated.

### Other processes involved in the response to stressful growth conditions

We showed that the XC-related NPQs is directly related to the decrease in Fv/Fm but it is likely that additional processes also contribute. The loss of PSII core function can also result in Fv/Fm decrease and is thought to participate to thermal dissipation as well^21^. Microalgal defenses against photo-oxidative stress also involve the cellular antioxidant network that was not investigated in this study. Carotenoids, besides their role in light harvesting, are important antioxidants^63,64^. Besides its role in NPQs, Dt may also act as a strong antioxidant. Lepetit, et al.^49^ observed that in diatoms grown under HL, a pool of Dt is found dissolved in the lipid matrix of the thylakoids, and probably does not participate to NPQ but is involved in preventing lipid peroxidation through direct scavenging of reactive O_2_ species. The remaining pool of Dt we observe after several hours’ recovery in the dark (Fig. 4) could be involved in ROS scavenging in thylakoid membranes. Moreover, we quantified huge amounts of echinenone, a carotenoid which is known to interact with the Orange-Carotenoid-Protein (OCP) to induce NPQ in cyanobacteria but, which role is still unknown in eukaryotic microalgae. In *T. lutea,* OCP was not found^28^. Additionally, echinenone does not decrease during dark recovery as Dt does (Figure S3). Echinenone may participate to photoprotection through direct ROS scavenging in a similar way as Dt molecules^49^. The antioxidant potential of echinenone has been demonstrated^65,66^ but its role in ROS scavenging remains to be confirmed.

### Echinenone versus Dt: contrasting dynamics

**Figure 6:**
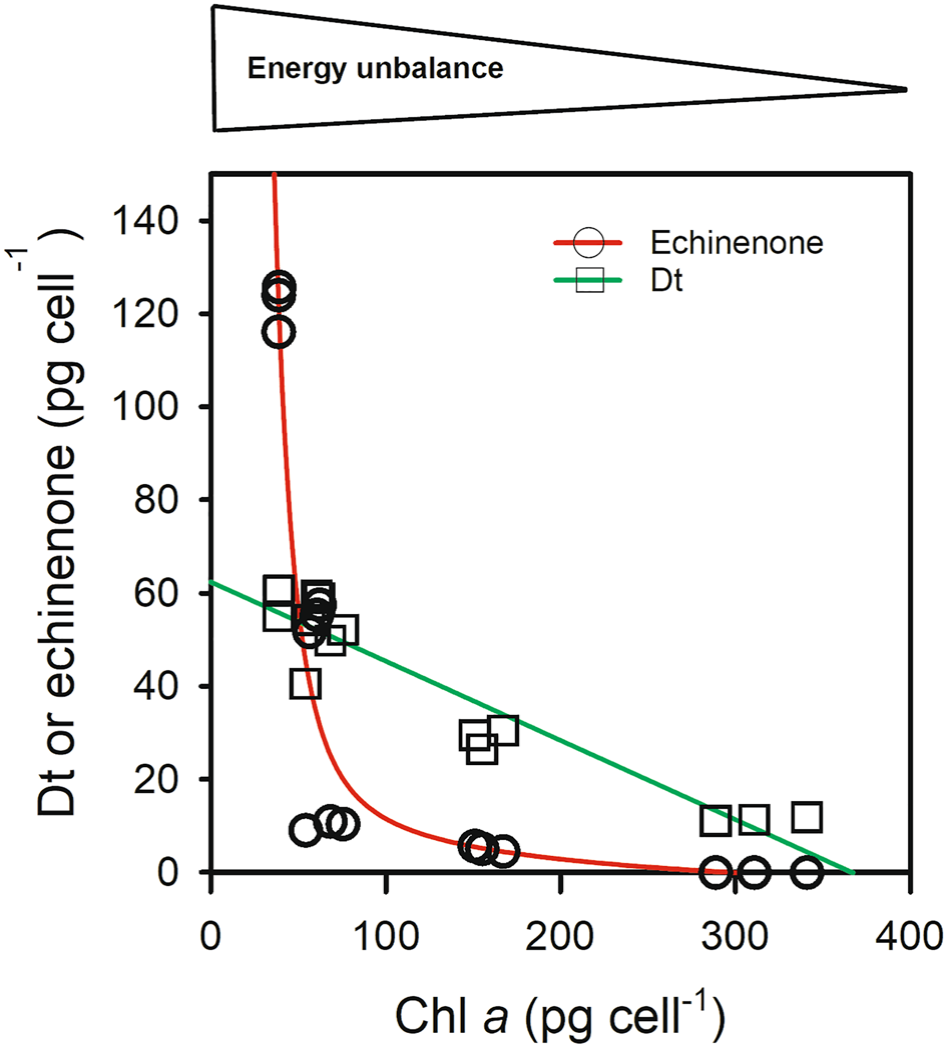
Relationship between Chl*a*/cell and Dt/cell (squares) and echinenone/cell (circles) in *T. lutea*. As energy unbalance increase (illustrated in the scale above the graph), Chl *a* per cell decreases and both diatoxanthin and echinenone increase.

In order to confirm this hypothesis, we further explored the changes in echinenone and Dt contents as a function of cell energy unbalance. As shown before^29^, echinenone was very abundant in starved cells (Table 1), as was Dt. However, it was almost absent under HL in nutrient replete cells. In order to illustrate the respective involvement of Dt and echinenone in the acclimation to growth conditions, we plotted the relationship between Chl *a*/cell and Dt and echinenone, respectively (Fig. 6). We found a linear relationship between Chl *a* and Dt, suggesting that XC-related NPQ and photoacclimation of cell specific absorption are regulated similarly. Echinenone behaved very differently: it slightly increased as Chl *a* decreased when the energy unbalance was moderate but drastically increased when conditions were getting worse. This behavior is consistent with the hypothesized role of echinenone mentioned above, i.e. when acclimation strategies of the cells (reduction of light absorption, dissipation of the excess absorbed light) are no longer sufficient, the ROS are likely to be scavenged by echinenone.

## Conclusion

In aquatic photosynthesis research, NPQ is often seen as a flexible mode of excess light energy dissipation in response to rapid changes in light climate which are inherent to water body mixing. Rapid NPQd was extensively described in diatoms and is involved in their ecological success, especially under extreme light climates^5,67,68^. However, NPQ is also a key process in microalgae long term acclimation to growth conditions^14,69^, and particularly under unfavorable conditions (nutrient starvation, low temperature, prolonged high irradiance). This long term sustained NPQ is poorly understood and the molecular mechanisms involved unknown (in particular specific LHCx proteins and/or specific Dt pool, the role of ΔpH, etc.). Also, we do not know how widespread NPQs is in the global ocean, but it certainly questions the use of adapted protocols (i.e. with extended NPQ relaxation period prior to measurement) in field studies, particularly in nutrient starved environments (oligotrophic waters) and situations (post-bloom).

### Supplementary Information

Supplementary Figures.

## Data Availability

All relevant data is contained within the manuscript: All datasets generated for this study are included in the manuscript and the Supplementary Files.
