# Supplementary material for: Sustained xanthophyll pigments-related photoprotective NPQ is involved in photoinhibition in the haptophyte Tisochrysis lutea
Source: Sci Rep. 2023 Sep 7;13:14694. doi: 10.1038/s41598-023-40298-z (PMC10484918; doi:10.1038/s41598-023-40298-z)
Supplement: Supplementary file 1 — Supplementary Figures. [file 41598_2023_40298_MOESM1_ESM.docx]

**Supplementary material**

**
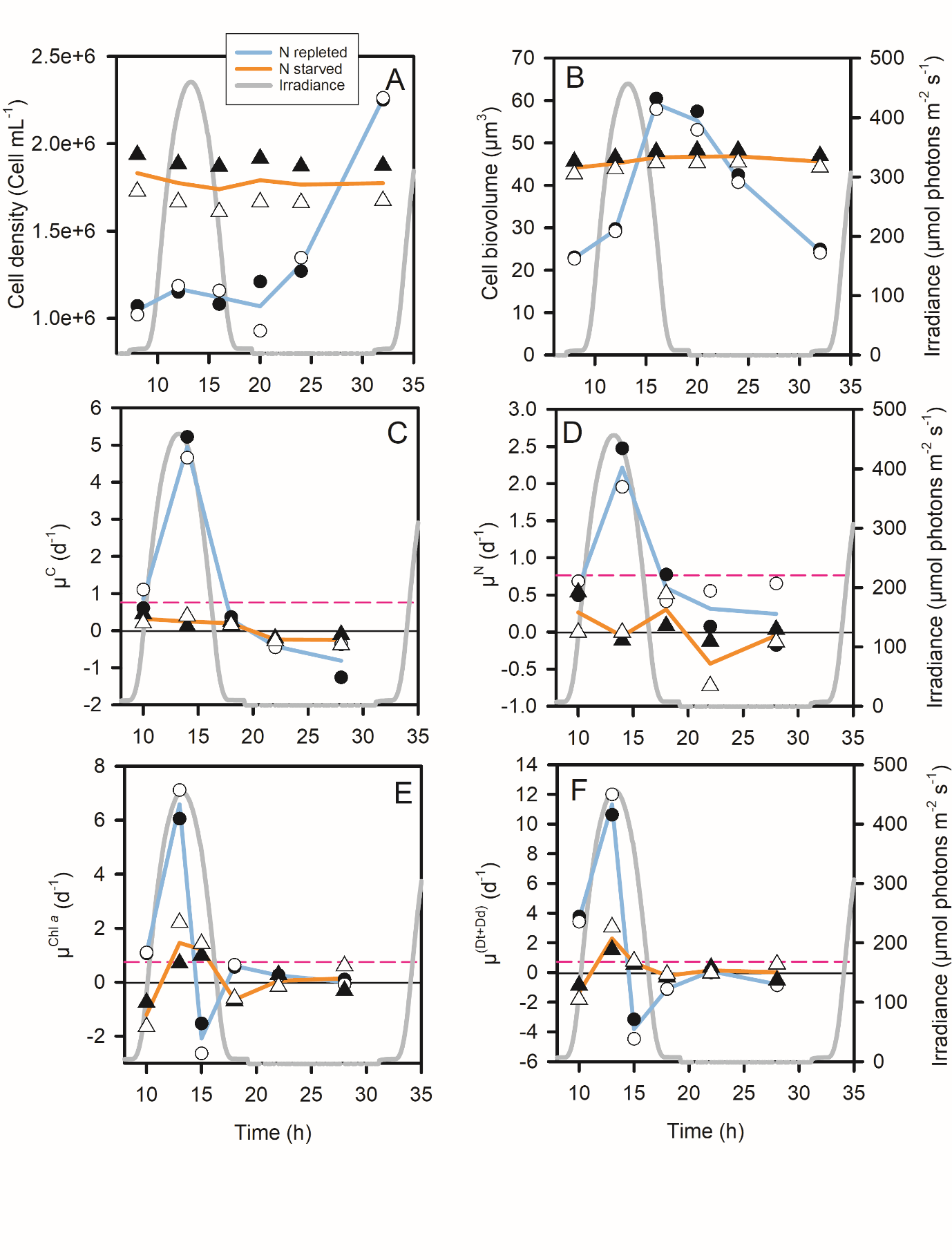
**

**Figure S1:** Time dependent change in mean cell biovolume (A), cell density (B), carbon specific growth rate (C), nitrogen specific growth rate (D), Chl *a* specific growth rate (E) and xanthophyll specific growth rate (F) during a light ⁄ dark cycle in N-sufficient (circles) and N-starved growth (triangles). The continuous grey line represents growth irradiance. The dashed red line represents the daily averaged cell division rate (0.77 d^-1^). In each graph, a line passes through the mean of the two replicates.

**
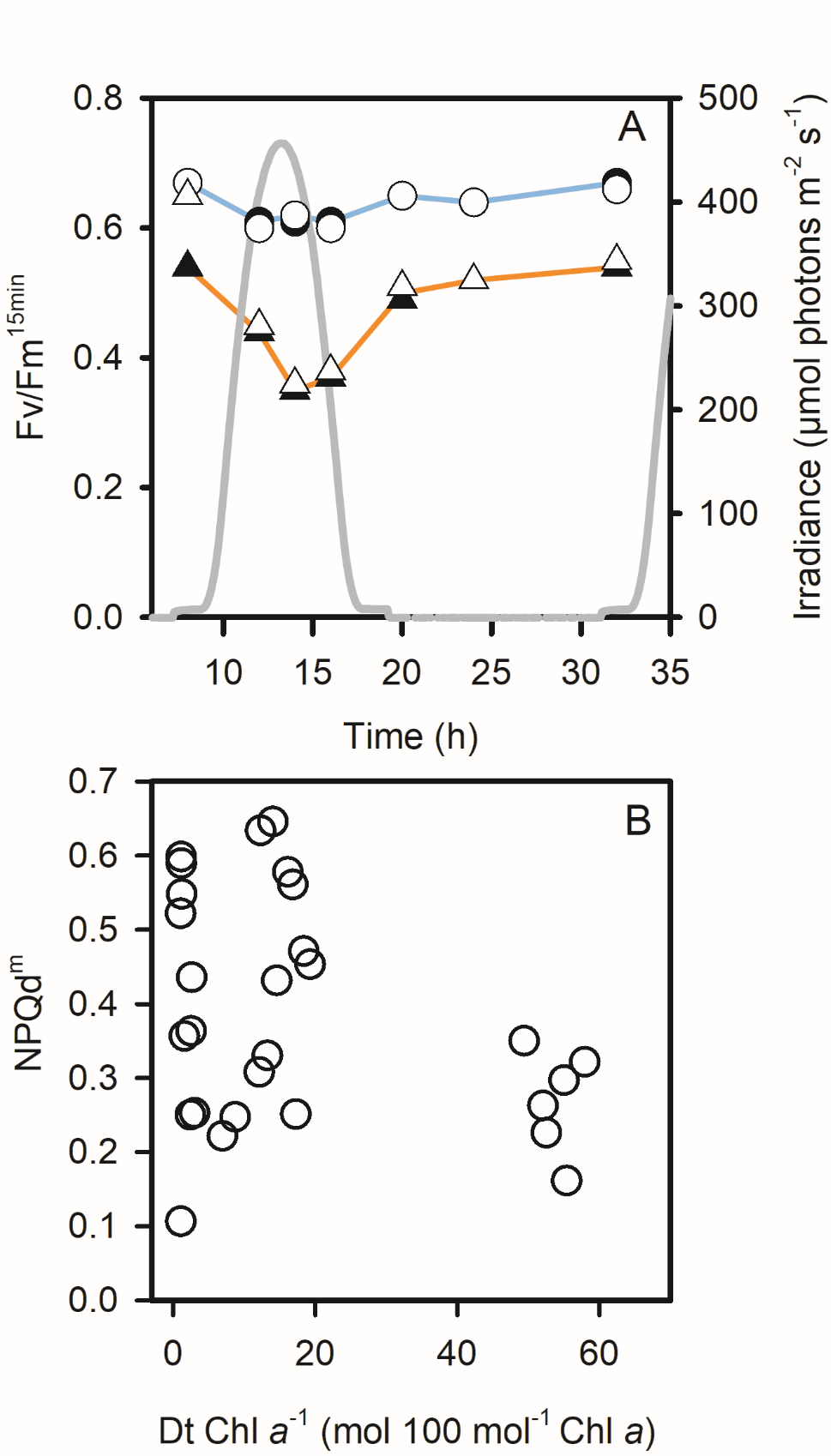
**

**Figure S2:** Time dependent change in Fv/Fm^15min^ (A) during a light ⁄ dark cycle in N-sufficient (circles) and N-starved growth (triangles) and relationship between NPQd^m^ and Dt/Chl *a* in N-sufficient (B). In A, the continuous grey line represents growth irradiance and a line passes through the mean of the two replicates.

**
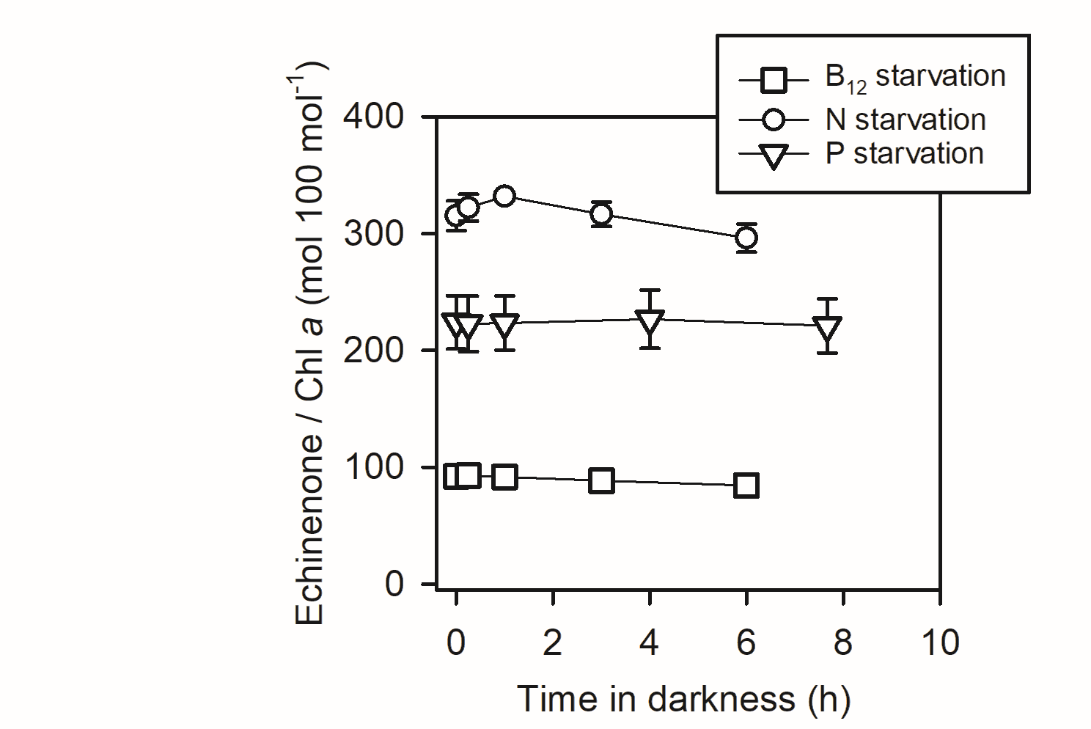
**

**Figure S3:** Darkness time dependent change in echinenone/Chl*a* in *T. lutea* cells under B_12_, N, P starvation (B)
